# HMGB1: The metabolic weapon in the arsenal of NK cells

**DOI:** 10.1080/23723556.2016.1175538

**Published:** 2016-04-15

**Authors:** Adelheid Cerwenka, Jürgen Kopitz, Peter Schirmacher, Wilfried Roth, Georg Gdynia

**Affiliations:** a German Cancer Research Center, Research Group Innate Immunity, Heidelberg, Germany; b Institute of Pathology, University of Heidelberg, Heidelberg, Germany; c Institute of Pathology, University Medical Center Mainz, Mainz, Germany; d German Cancer Research Center, Clinical Cooperation Unit Molecular Tumor Pathology, Heidelberg, Germany

**Keywords:** Cancer, cell death, HMGB1, immunotherapy, innate immunity, metabolism, Natural Killer Cells, NK cells

## Abstract

Targeting tumor glycolysis would hit the main energy source of cancer. We show that natural killer (NK) cells pursue this strategy by employing high mobility group box 1 (HMGB1) protein—a well-known proinflammatory cytokine—to specifically target glycolysis in cancer cells. This opens up new perspectives for cancer immunotherapy.

Natural killer (NK) cells can eliminate cancer cells within minutes.^1^ Therefore, mimicking or stimulating this most effective innate immune response to cancer is a major goal of different immunotherapy approaches.^2^ Identification of new components and/or strategies of the NK cell lytic program could be highly beneficial for the design of novel treatment strategies for cancer patients. Recently, we described a new link between tumor elimination by innate immunity and metabolism.^3^ It is well known that high concentrations of high mobility group box 1 (HMGB1) protein are secreted from NK cells upon stimulation.^4^ ELISA experiments performed in our laboratory revealed that secreted HMGB1 reaches concentrations up to 1,000-fold higher than those of interferon-gamma (IFNγ) upon stimulation of the natural cytotoxicity receptor (NCR) Nkp30 (unpublished data). However, HMGB1 was not previously linked with direct cytotoxicity toward cancer cells.

HMGB1 is a cytokine that on one hand promotes inflammation and on the other hand regulates the energy metabolism of cells.^5^ We previously showed that recombinant HMGB1 induces a new type of necrotic cell death accompanied by giant swollen mitochondria.^6^ In a more recent study we isolated HMGB1 from the cytotoxic granules of NK cells and tested its cytotoxic potential against colon cancer cells.^3^ Radioactively labeled HMGB1 rapidly penetrated the cells, most probably via its Tat-protein-like domain, and accumulated in all cellular compartments. The striking perturbation in mitochondrial morphology with giant hollow mitochondria with rudimentary cristae and elevated lactate levels after treatment with HMGB1 resembled the impairment of oxygen-dependent energy metabolism that can be observed in human mitochondrial diseases. Consistent with these findings, luciferase assays revealed a decrease in ATP levels within 72 h. Mitochondrial respiration was decreased in different colon cancer cell lines and in organotypic cultures obtained from fresh human surgical colorectal cancer specimens. The oxygen consumption of the mitochondrial respiratory chain, as measured by high-resolution respirometry (Oroboros 1 oxygraph system), revealed a differentiated phenotype of colorectal cancer cells upon HMGB1 treatment: respiration ceased in cells that were highly sensitive to HMGB1 whereas it was not affected in resistant cells such as the HT29 colon carcinoma cell line. These resistant cells increased glutamine oxidation as measured by production of glutamine-derived CO_2_ and thus sustained ATP production from mitochondrial respiration. However, immunohistochemical staining of malic enzyme 1 (ME1) that facilitates glutamine breakdown in 1,260 colorectal carcinoma tissue specimens showed that glutaminolysis was important for locoregional tumor growth but not for metastasis formation. This finding suggests that in the long run the detection of cancer cells with no need for aerobic respiration might be associated with patient survival.

Indeed, isotope experiments revealed that upon HMGB1 treatment colon cancer cells were forced to switch to anaerobic glycolysis (breakdown of glucose to lactate). Cells that were depleted of mitochondria (rho zero cells), and thus perfectly adapted to anaerobic energy metabolism, were resistant to HMGB1. Consequently, NK cell-mediated cell death induced via HMGB1 particularly targets cancer cells that rely on oxygen-dependent energy metabolism.

Solid tumors are composed of a mixture of normoxic and hypoxic/anoxic cancer cells. The latter can be visualized by immunohistochemical staining of pimonidazole or its derivates (administered to the patient before surgical resection of the tumor) that accumulate in anoxic cells. It is well known that these cells are the preferential source of metastases and are resistant to (radio-)chemotherapy, thus determining the patient outcome.^7^ The experiments in our previous work showed that HMGB1-mediated killing of cancer cells by NK cells (with up to 160 nM HMGB1) is restricted to normoxic cancer cells that still rely on aerobic respiration (Fig. 1). However, the *in vivo* concentration of HMGB1 at the effector-target interface might be higher than that used in our experiments. Thus, higher concentrations of HMGB1 might also kill anoxic cancer cells that rely solely on anaerobic glycolysis. These cells show a specific strategy for macromolecule synthesis: inhibition of the pyruvate kinase (PK) reaction by phosphotyrosine peptides shunts glucose carbons to anabolic pathways.^8^ In fact, we have shown that HMGB1 polyphosphorylated on its tyrosine residues at the B Box domain inhibits the high-affinity pyruvate kinase isoform, subsequently blocking glucose-driven respiration. However, all experiments were performed under normoxic cell culture conditions. To mimic the low/depleted oxygen environment found in solid tumors, HMGB1 anticancer cytotoxicity should be assessed under hypoxia/anoxia. Preliminary data from our laboratory suggest that HMGB1 also has the potential to kill anoxic cancer cells. One reason for this could be that cancer cells operating without oxygen have to switch between low (supplying anabolic pathways, i.e., pentose phosphate shunt) and high (fueling anaerobic energy metabolism) PK activity and HMGB1 would impair this plasticity.

**Figure 1.**
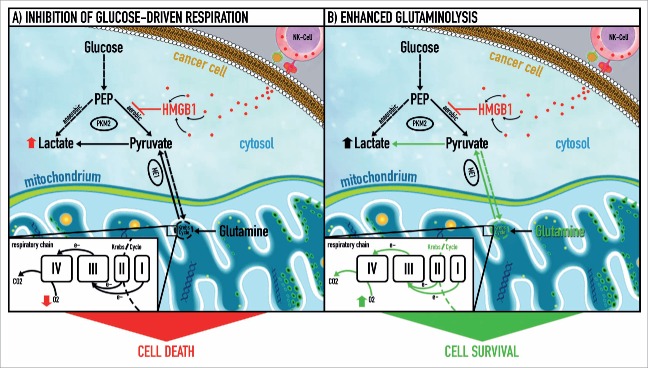
NK cell-derived HMGB1 controls glycolysis and aerobic respiration by allosteric inhibition of tetrameric pyruvate kinase M2. (1) High mobility group box 1 (HMGB1) protein induces a metabolic type of cell death in cancer cells via inhibition of tetrameric pyruvate kinase (PK) M2 and subsequent blockage of glucose-driven respiration. Thus, HMGB1 rapidly forces cancer cells to rely on glycolysis (employing dimeric PK M2) as the only remaining source of energy production. (2) A subgroup of cancer cells can evade HMGB1-induced metabolic cell death by switching to glutamine breakdown and thus preserving electron flux through the respiratory chain. The latter is coupled to oxidative phosphorylation and provides an alternative energy source that compensates for the energy deficit caused by inhibition of glucose-dependent aerobic respiration through HMGB1. This metabolic plasticity allows cancer cells to resist HMGB1-induced metabolic cell death. e^−^, electron flow; ME I-IV, malic enzyme complex I-IV; PEP, phosphoenolpyruvate; PK M2, pyruvate kinase isoform M2.

One implication of these findings is the necessity to estimate the number of anoxic cancer cells in a solid tumor. An approach that is daily routine in microbiology, where bacteria are classified as aerobic or anaerobic strains and then differentially treated, might be also beneficial in cancer therapy. The regulation of glycolytic enzymes, in particular PK activity, under hypoxic/anoxic conditions is a suitable indicator for anaerobic cancer cell growth.^9,10^ By starting a spin-off company we intend to deliver a test that could be applied for diagnostic use by advising the clinician whether there is a high number of anoxic cancer cells in a given tumor sample.

The isolation and purification of HMGB1 as described in our work can be scaled up for industrial production. The NK cell line NK-92 is well suited for culture in bioreactors. This enables cost-effective production of significant amounts of HMGB1 with potent anticancer activity. HMGB1 then could be used for anticancer immunotherapy; however, potential side effects should first be ruled out in toxicologic studies.
